# Culturally acceptable fermented grain may improve gut health in South African postpartum mothers in a randomised trial

**DOI:** 10.1017/S0007114526106862

**Published:** 2026-03-25

**Authors:** Anna-Ursula Happel, Katie M. Strobel, Obakeng Jona, Janine Fredericks, Brian Kullin, Brandon Perumaul, Adeebah Rakiep, Marjanne Senekal, Sonia Malczyk, Johanna Helena Nel, Marijke A. Fagan-Endres, Jo-Ann S. Passmore, Heather B. Jaspan

**Affiliations:** 1 Department of Pathology, University of Cape Town, Cape Town, South Africa; 2 Institute of Infectious Disease and Molecular Medicine (IDM), University of Cape Town, Cape Town, South Africa; 3 Seattle Children’s Research Institute, Seattle, USA; 4 University of Washington, Seattle, USA; 5 Department of Chemical Engineering, University of Cape Town, Cape Town, South Africa; 6 Department of Human Biology, University of Cape Town, Cape Town, South Africa; 7 Department of Logistics, Stellenbosch University, Stellenbosch, South Africa; 8 National Health Laboratory Service, Cape Town, South Africa

**Keywords:** Diet, Gut health, Fermented foods, Maternal health, South Africa

## Abstract

Optimising nutrition during lactation is critical for the mother and infant. The relationship between fermented food consumption and the mother’s gut microbiota and nutritional and inflammation status is unknown. Mageu is a fermented grain commonly consumed in Southern Africa. We randomised South African mothers to consume a live-culture mageu, pasteurised store-bought mageu or no mageu from 4 to 10 weeks postpartum. Clinical and dietary data, stool microbiota and nutritional and inflammatory biomarkers were assessed until week 15. Plant protein intake was higher among mageu users than non-users. Live-culture mageu increased gut *α*-diversity from weeks 4 to 10. Circulating ferritin was lower among live-culture mageu users at week 10 compared with non-users. In systems analyses, mageu intake was associated with distinct bacterial, inflammatory and nutritional signatures, primarily driven by interleukin (IL)-6, ferritin, soluble transferrin receptor and *Eubacterium hallii*. These results suggest that mageu has benefits for lactating mothers’ gut health and, therefore, possibly their infants.

Maternal health and nutrition are critical to preventing morbidities for both the mother and her child^(1)^. In South Africa, 70 % of reproductive-aged women are overweight, almost 50 % are hypertensive, 30 % are anaemic and one-third of children < 5 years old are considered clinically stunted^(2)^. South Africa, like many countries in Africa, has significant economic disparities. The South African General Household Survey conducted in 2021 indicated that 21 % of households had inadequate access to food, with two-thirds of affected households being in urban areas^(3)^. It has also been estimated that ~50 % of the general South African population was consuming low-nutritional-diversity meals, with diets based predominantly on starches^(4)^.

It is well established that diet is a driving factor in gut microbiome composition and function^(5,6)^, and short-term dietary interventions have been shown to impact the gut microbiome rapidly^(7–9)^. The gut microbiome is also known to impact systemic inflammation^(8,9)^, an underlying cause of many non-communicable diseases in mothers. Chronic inflammation has also been associated with stunting in infants^(10)^. Thus, a key question is whether diet modification using locally acceptable fermented foods is an avenue to improve nutrition and systemic inflammatory status in both lactating South African mothers and their infants.

Throughout human history, fermentation has served to preserve and process foods, and there is a growing realisation that fermented foods have both nutritional and other health benefits^(8,11–13)^. Transforming original food substrates through fermentation leads to an extended shelf life, the formation or increase of bioavailable end-products, including vitamins, amino acid derivatives and organic acids, and may remove or reduce food toxins^(14)^. This serves important purposes in regions like Southern Africa, where food security is low and access to refrigeration, electricity and running water is limited^(3)^. Significant associations have been reported between the consumption of fermented food and clinical outcomes, including improved weight maintenance^(11)^, reductions in the risk of cardiovascular disease, type 2 diabetes, gestational diabetes and overall mortality^(12,13)^. These studies were however primarily conducted in the global North, while data from Africa are scarce. Meanwhile, observational and interventional studies found fermented food consumption to have a beneficial impact on the gut microbiota and immunity^(8,15)^. In agreement, a meta-analysis of randomised controlled trials that evaluated the effect of fermented foods consumption on systemic inflammation found that fermented food intake decreased levels of tumor necrosis factor (TNF)-*α*, a common biomarker of inflammation^(16)^.

As fermentation is widely practised in Southern Africa as a household-level technology to preserve foods, a highly fermented food diet is likely to offer a culturally acceptable and easily implementable intervention to improve maternal health outcomes. Mageu is a fermented, non-alcoholic cereal-based sour porridge, popular throughout Southern Africa^(17–19)^. Traditional preparations of mageu include making a maize-porridge with a natural inoculum source, such as wheat, subsequently added to initiate fermentation with bacteria including *Lactobacillus delbrueckii*, *Lactiplantibacillus plantarum*, *Streptococcus thermophilus* and *Lactococcus lactis*^(20)^. Knowledge of traditional recipes of mageu is common in older generations, while large commercial production of mageu now exists in Southern Africa^(21)^. However, commercial mageu is pasteurised, which increases the shelf life of the product further but also reduces the viability of the live organisms that were present during fermentation^(21)^. It is unclear whether the consumption of fermented foods that contain live microorganisms at the time of consumption would be superior to the consumption of pasteurised fermented foods (without viable organisms) on long-term health benefits and the gut microbiota and inflammatory status of consumers.

As most clinical evidence for fermented food’s impact on health comes from high-income countries, we tested the hypothesis that fermented mageu improves gut microbiota in a pilot randomised controlled trial among postpartum, lactating South African mothers. We compared gut microbial changes, nutritional metrics and systemic inflammation between lactating mothers consuming unpasteurised, live-culture mageu (LCM, developed as part of this project) *v*. pasteurised, store-bought mageu (SBM) *v*. no mageu for 6 weeks postpartum.

## Methods

### Interventional product preparation

LCM was produced by the Centre for Bioprocess Engineering Research at the University of Cape Town, in line with the regulations stipulated in South African National Standards 1199:2011 ‘The production of mageu’. For each batch, a 10 wt% maize meal in water suspension was prepared and cooked at 90–100°C for 15 min. The porridge was cooled to 35–40°C, after which wheat flour was added as the inoculum source (10 g: 1 l porridge). The LCM taste was also adapted at this point using sugar (20 g: 1 l porridge), so that the final product matched the SBM flavour (Number 1 Mageu Cream Flavor, RCL FOODS) and nutritional properties as closely as possible. The porridge was fermented at 37°C for 48 h. The quality and nutritional characteristics of the final product were externally verified through an accredited nutritional, microbial and pathogen testing laboratory (Microchem Specialised Lab Services).

### Interventional product quality control

Quality control evaluations were performed for each batch of LCM and SBM according to South African National Standards 1199:2011. Mageu dilutions were spread onto MacConkey agar plates (Millipore HG00002·500, made according to manufacturer’s instructions), incubated aerobically for 24 h at 37°C and assessed for the presence of *Escherichia coli*. To identify anaerobic spore-forming bacteria (*Clostridium* spp.), mageu dilutions were incubated at 80°C for 12 min and then plated onto supplemented brain heart infusion (BHI) agar (37 g/l BHI, 5 g/l yeast extract, 0·5 g/l cysteine-hydrochloride, 5 mg/l haemin, 1 mg/l menadione and 15 g/l agar) and incubated anaerobically for 48 h at 37°C. Bacterial colonies were subcultured onto fresh BHI agar plates at 37°C for 24 h aerobically to distinguish between *Clostridium* spp. *v*. aerobic spore formers. Colonies of interest on MacConkey agar and oxygen-sensitive colonies on BHI agar were counted to determine the number of colony-forming units (CFU/ml). Colonies were typed by PCR and sequencing of the 16S rRNA gene. A previously described colony PCR protocol^(22)^ was used with the universal 16S rRNA gene primers 27F and 1492R^(23)^. Products were Sanger sequenced (Inqaba Biotec) using the 907R primer and the resulting sequences classified by comparing to the National Center for Biotechnology Information 16S rRNA gene database using the Basic Local Alignment Search Tool algorithm^(24)^.

### Study participants

Mothers were recruited between June 2022 and June 2023 from the Midwife Obstetric Unit in Khayelitsha, an informal settlement in urban Cape Town, South Africa. Maternal eligibility criteria included having had a negative HIV test in the past 6 weeks, being aged between 18 and 50 years, electing to breastfeed, having access to a refrigerator and electricity at home, being willing and able to consent and be randomised, and having delivered at full-term, average-for-gestational-age birth weight infant within the past 10 d. Exclusion criteria included complications during pregnancy and delivery (i.e. gestational diabetes, body-mass-index (BMI) > 40 prior to pregnancy, chorioamnionitis or eclampsia), active tuberculosis or other infectious diseases, or administration of antibiotics, commercial probiotics, prebiotics or immunoregulatory products. A screening questionnaire was administered to assess maternal fermented food consumption, and women who had consumed > 5 servings of fermented foods in the week prior to delivery were excluded. Based on previous work^(8)^, serving sizes of the items included in the questionnaire were set as follows: mageu, amasi (a milk-based traditional fermented food), buttermilk and yoghurt: 1 serving = 250 ml/g; atchar (pickles): 1 serving = 62·5 ml/45 g; homemade fermented porridge: 1 serving = 250 ml/g; and traditional/home brewed beer: 1 serving = 250 ml. Administration of the screening questionnaire involved showing potential participants a picture card of each item and asking them whether they had consumed it in the week prior to giving birth. The amount of the indicated items consumed was quantified by determining the frequency consumed in that week and the typical serving size, which was then converted to a total number of servings consumed per week. Quantification of serving sizes was done using applicable line sketches and actual containers.

### Randomisation and masking

At enrolment, women were randomly assigned 1:1:1 to the SBM, LCM or no mageu group. Participants were assigned using the Excel (Microsoft) function ‘randomise’, and the randomisation list was issued to the pharmacist. Participants were blinded to the allocated intervention arm, except for the no mageu group. All personnel involved in clinical and laboratory testing and investigators were masked to group assignments, except for the dietitians analysing the dietary data and the pharmacists.

### Study procedures

At the enrolment visit, all women were requested to refrain from consuming mageu and other fermented foods for a 3-week washout period (including consumption of < 500 ml of amasi, yoghurt or buttermilk per week). All women also received dietary counselling to maintain quality protein and calcium intake throughout the study. Information was collected about maternal socio-economic and demographic indicators, household food insufficiency and insecurity and dietary intake. Data were entered directly into standardised case report forms on a REDCap database^(25)^. A physical exam was performed, including weight and height measurements. All women were encouraged to exclusively breastfeed their infants for 6 months, in accordance with the current WHO guidelines^(26)^. At 4 weeks postpartum, women returned to the clinic for sample collection and questionnaires and to receive their randomised intervention (either SBM, LCM or no mageu). The mageu groups were blinded as to the type of mageu women would be receiving, and women were instructed to consume one bottle of 500 ml daily for 6 weeks. Women in the no mageu group were instructed to continue their regular diet. All women were counselled about appropriate caloric intake to ensure that the addition of mageu to their diets did not result in increased calorie intake and weight gain, to continue consuming no more than 500 ml of amasi/yoghurt/buttermilk per week and to refrain from the consumption of other fermented foods for the duration of the study. Since the shelf life of opened SBM was 4 d (as per manufacturer’s instructions, SBM was opened to transfer to fresh packaging as part of blinding protocol), a 3–4-d supply was provided to each participant randomised to SBM and LCM. Twice weekly, the study driver delivered the next few days’ mageu directly to participants’ homes at 4°C. Participants were asked to store the mageu in their household refrigerators. Additional clinic visits were at 7 weeks, 10 weeks (end of 6-week intervention) and 15 weeks (5 weeks post-intervention).

### Monitoring of intervention

To monitor compliance with the intervention (mageu intake: yes/no, and if yes, amount), as well as daily intake from ten food grsoups, participants completed daily monitoring sheets for the duration of the 6-week intervention. Participants were trained to mark food groups from which items had been consumed using a food photograph guide developed for these purposes. The ten food groups were aligned with the FAO food group guide for the calculation of dietary diversity for adult women^(27)^ and included grains, roots and tubers, pulses, nuts and seeds, dairy products, flesh foods, eggs, dark green leafy vegetables, other vitamin A-rich fruits and vegetables, other vegetables and other fruit. Dietary diversity was calculated as a score out of 10 as stipulated by the FAO (2021). Monitoring sheets were reviewed at weeks 7 and 10 by the dietitian to assess adherence. Additional measures of adherence included bottle returns to the pharmacy.

### Clinical assessments

At each visit, maternal health outcomes, including adverse events (AEs), were collected using standardised case report forms. The study clinician assessed AE severity and relatedness using the Division of AIDS Table for Grading the Severity of Adult and Pediatric Adverse Events, Corrected Version 2.1^(28)^. Maternal height and weight were measured at each time point to calculate BMI. The use of concomitant medication was captured on daily monitoring sheets.

### Nutritional assessments

Dietary assessment for estimation of total energy, macronutrient (carbohydrates, protein, fat), fibre and micronutrient (minerals, antioxidant compounds and B vitamins) intake involved three face-to-face 24-h recalls administered using the multiple pass method^(29)^ at weeks 4, 7 and 10. Serving size was estimated using a booklet adapted from the Dietary Assessment and Education Kit^(30)^. The 24-h recall data were analysed using the South African Food Composition Tables^(31)^. The three 24-h recalls were used to calculate the mean energy and nutrient intake adjusted for intra-person variability using the Institute of Medicine method^(32)^ over the 6-week intervention period.

### Specimen collection

Stool samples from women were collected in sterile specimen collection cups at each visit and transported to the laboratory at 2–8°C and stored at −80°C. Six ml of maternal whole blood was collected at matching time points both in Sodium heparin and serum separator tubes. The whole blood was centrifuged at room temperature for 10 min at 344 g and aliquoted, and plasma and serum were stored at −80°C for testing of inflammation and nutritional biomarkers.

### Nucleic acid extraction, 16S rRNA gene amplification and sequencing of mageu and maternal stool

DNA was extracted from 400 μl of mageu stored in a 1:1 ratio in Primestore^®^ Molecular Transport Medium (Longhorn Vaccines & Diagnostics LLC) and from 200 mg of dry maternal stool collected at weeks 4, 10 and 15, using the DNeasy Powersoil Pro kit (Qiagen) following the manufacturer’s protocol, with mechanical disruption by bead-beating at 50 Hz for 10 min (Qiagen TissueLyser LT). Positive controls (bacterial mock community HM-280, BEI) and negative controls (nuclease-free water) were included for cross-contamination filtering and error rate modelling. DNA was amplified in triplicate using primers designed to span the V3–V4 region of the 16S rRNA gene (357F/806R), as described previously^(33)^. Amplicon libraries were purified using AMPure XP beads (Beckman Coulter), quantitated via Quant-iT dsDNA High Sensitivity Assay (ThermoFisher) and pooled in equal mass quantities. Paired-end sequencing was performed on an Illumina MiSeq platform using V3 600-cycle kits.

### Quantification of total bacterial abundance in mageu samples

The total 16S rRNA gene copies/μl extracted gDNA were measured as previously described^(34)^. Standards from 1 × 10^8^ to ten copies, a no-template control and samples were run in duplicate using the SsoAdvanced Universal Probes Supermix (Bio-Rad) and acquired on a QuantStudio 7 Flex Real-Time PCR System (Applied Biosystems). Samples were re-run if the duplicate reactions differed by more than0·3 Ct cycles, or if the Ct values fell outside the dynamic range of the standard curve. Samples with less than ten copies were deemed to be below the lower limit of detection of the assay.

### Measurement of gut and peripheral inflammation and nutritional biomarkers

Concentrations of interleukin (IL)-1*β*, IL-6 and TNF-*α* were quantified in undiluted maternal plasma collected at weeks 4 and 10, using a Millipore Milliplex Human High Sensitivity T Cell Luminex Panel (Premixed 13-plex; HSTCMAG28SPMX13), as per manufacturer’s recommendations. Data were acquired using the Bio-Plex™ Suspension Array Reader (Bio-Rad Laboratories Inc^®^), and a 5PL regression line was used to determine the cytokine concentrations from the standard curves using the Bio-Plex™ manager software.

At weeks 4 and 10, lipocalin-2 (R&D Biotechne Human Lipocalin-2/Neutrophil Gelatinase–Associated Lipocalin Quantikine) and myeloperoxidase (R&D Biotechne Human Myeloperoxidase Quantikine) concentrations in maternal plasma, and faecal calprotectin (R&D Biotechne Human S100A8/S100A9 Heterodimer Quantikine) in maternal faecal extracts were measured by ELISA. The faecal extract was derived from 30 mg of dry maternal stool, as per kit instructions. The protein concentration in each stool sample was measured using a NanoDrop Spectrophotometer (ThermoFisher) at A280 nm, and 30 μg of total protein per sample was used. For all ELISA and multiplex assays, intra- and inter-plate controls were included, and CV ≤ 20 % were deemed acceptable. Maternal plasma collected at weeks 4 and 10 was evaluated for soluble transferrin receptor (sTfR, Fe sufficiency), retinol binding protein 4 (RBP4, indicator of vitamin A and protein status), thyroglobulin (Tg, iodine status), C-reactive protein (CRP) and *α*−1-acid glycoprotein (systemic inflammation) using the Q-Plex Human Micronutrient array (Quansys Biosciences). 25-Hydroxy vitamin D (25(OH)VitD_3_), vitamin B_12_, ferritin and Fe levels were measured in maternal serum collected at weeks 4 and 10 by the local National Health Laboratory Service Pathology lab in Cape Town, South Africa.

### Justification of sample size

The primary outcome was the change in maternal stool *α*-diversity using Shannon’s or Faith’s phylogenetic diversity (PD) index, from the start to the end of the intervention (week 4 to week 10). The primary outcome was assessed in the intention-to-treat population (including all women who were randomised). Secondary outcomes included cross-sectional differences in maternal gut microbiota *α* and *β* diversity and bacterial taxa at weeks 10 and 15, and changes in systemic inflammatory and nutritional markers from week 4 to week 10. While this was a pilot trial, we estimated that we would have 80 % power to detect a 17 % difference in stool *α*-diversity with a sample size of 15 women per group based on a preliminary analysis of 69 stool samples from non-pregnant, reproductive age women in Cape Town who had a mean gut microbiota Shannon index of 3·48 (SD 0·56).

### Data analysis

All sequence data processing, classification and amplicon sequence variant calling were performed using the Divisive Amplicon Denoising Algorithm 2 package (version 1.32.0)^(35)^ within the R framework (version 4.4.0). Amplicon sequence variants were taxonomically classified using an updated version of the Silva training set version 132^(36)^, available at https://github.com/itsmisterbrown/updated_16S_dbs. Run-specific contamination filtering was performed via decontam (version 1.24.0)^(37)^, and samples with fewer than 1000 filtered and annotated reads were discarded. The phyloseq (version 1.48.0)^(38)^, picante (version 1.8.2)^(39)^, ape (version 5.8)^(40)^, Deseq2 (version 1.44.0)^(41)^, microbiome (version 1.26.0)^(42)^ and vegan (version 2.6.6.1)^(43)^ packages were used for ecological analyses of bacterial communities. Inter-community distance was assessed using Bray–Curtis distance of relative abundance transformed abundance estimates. *α*-diversity was calculated using Shannon and Faith’s PD index. Because microbiota data are compositional, we employed centred log ratio (CLR) transformation^(44)^ for the differential abundance regression analysis. Taxa were agglomerated to the species level or the lowest taxonomic annotation. Statistical tests used include permutational ANOVA (or PERMANOVA) for comparisons among multiple (> 2) groups and Wilcoxon rank-sum tests for comparisons among two groups. For longitudinal analysis of diversity and CLR-transforsmed relative abundances from weeks 4 to 15, we utilised linear mixed models accounting for repeated measures using the lmer package in R (version 3.1.3)^(45)^. All taxa were eligible for longitudinal testing. Benjamini–Hochberg correction was utilised for multiple comparisons. Species-level presence/absence matrices were created from the phyloseq object aggregated at the species level. To identify taxa uniquely associated with the LCM group, we examined each visit-specific subset (weeks 10 and 15) and defined ‘LCM-unique’ taxa as those present in at least one LCM participant and absent in all participants of the SBM and no mageu groups. For each LCM-unique species, we calculated prevalence within the LCM group as the proportion of LCM participants in whom the species was found. The Data Integration Analysis for Biomarker discovery using Latent cOmponents (DIABLO) framework, as part of the mixOmics R Bioconductor package (version 6.25.1)^(46)^, was used for multi-omics analyses integrating the microbial, nutrition and inflammation data. Although this was a pilot trial, *P*-values were adjusted for multiple comparisons using Dunn’s test.^(47)^

## Results

**Figure 1.**
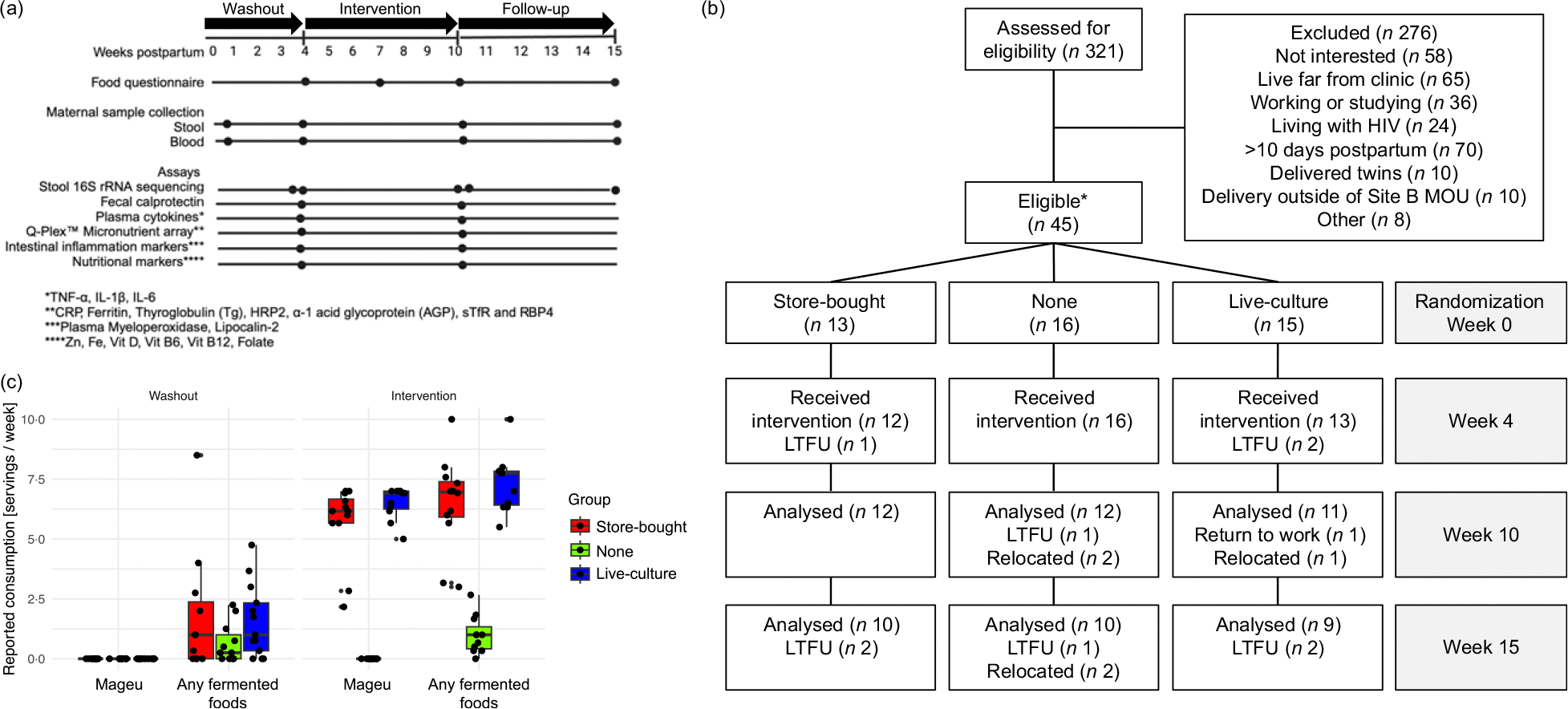
Overview of the Mamisa randomised controlled trial. (a) Study scheme showing the timeline, sample type collection and corresponding experimental platforms that are presented in this manuscript. (b) Consort flow diagram for participant enrolment, allocation, follow-up and analysis for each of the randomisation groups reflecting the time points that are presented in this manuscript. *One participant from the store-bought mageu group was excluded from the analysis as she acquired HIV after enrolment. LTFU = loss to follow-up. (c) Reported consumption of mageu and any fermented foods (including mageu and other fermented foods) by randomisation group during the washout (weeks 0–4) and intervention (weeks 4–10) periods. Median and interquartile ranges are displayed.

To examine the effect of a culturally acceptable fermented grain, mageu, on postpartum maternal health, 321 generally healthy women were screened for eligibility, of which forty-five were randomised to one of three arms: SBM (*n* 14), no mageu (*n* 16) or LCM (*n* 15) (Figure 1(a) and (b)). One participant sfrom the SBM group was excluded as she acquired HIV during follow-up, resulting in thirteen participants included in the SBM group for the primary analysis. The median maternal age was 27 (IQR 23–33) years (Table 1). About half of the women lived in informal housing, and most were unemployed during the study period. The median BMI at week 4 postpartum (after washout period, pre-intervention) was 29·6 (IQR 26·6–34·9), with almost half (19/41, 46·3 %) being obese (BMI >= 30) and the remainder mostly being overweight (17/41, 41·5 %, BMI >= 25−30).

There were twelve women each in the no mageu and SBM groups and eleven women in the LCM group available for inclusion at the primary endpoint visit (week 10; Figure 1(b)). Reasons for non-inclusion were relocation (*n* 3), return to work (*n* 1) and loss to follow-up (*n* 4). Participants in the SBM and LCM groups increased their mageu consumption during the 6-week intervention period in line witsh the protocol, as determined from the daily monitoring sheets. While all participants reported no consumption of mageu during the washout period (weeks 0–4), women assigned to SBM reported consumption of an average of 6·0 (SD 1·16) servings of 500 ml each per week. Similarly, the LCM group reported mageu consumption of an average of 6·5 servings of 500 ml (SD 0·67) per week during the intervention period (weeks 4–10) (Figure 1(c)). Consumption of other fermented foods was rare, with an average of < 2 servings per week being reported by women randomised to all groups (Figure 1(c)).

### Live-culture mageu had a higher bacterial load and a more complex bacterial community than store-bought mageu

**Figure 2.**
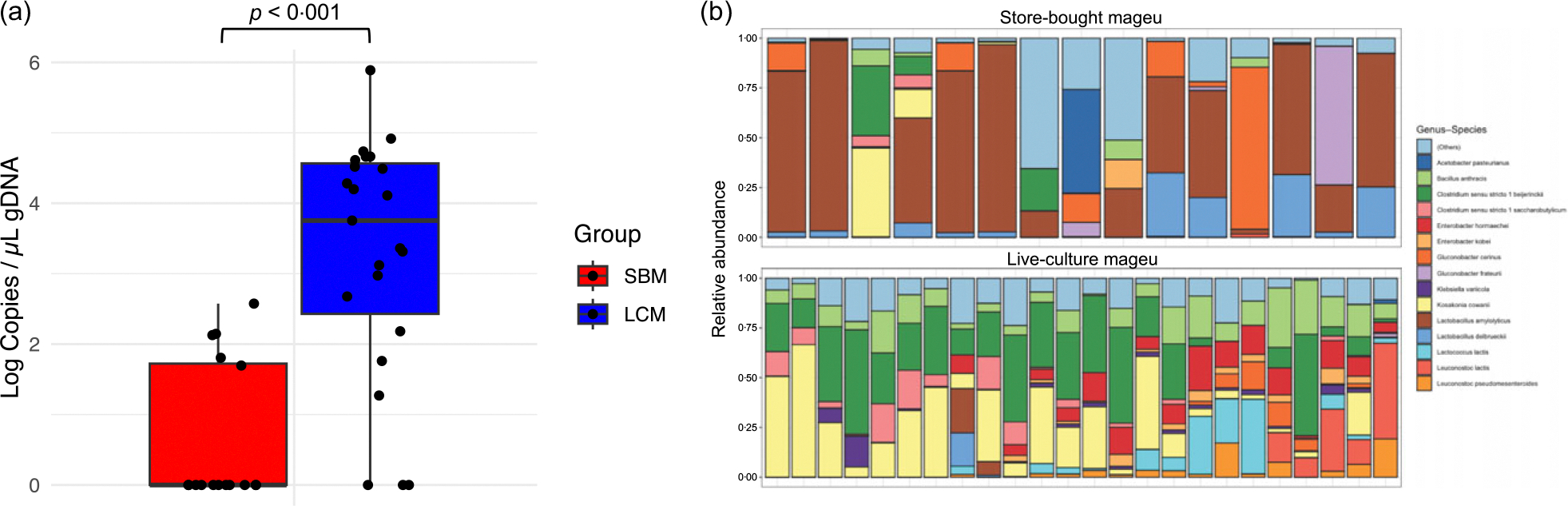
Molecular analysis of magseu. (a) Absolute 16S rRNA gene copy number in store-bought mageu (SBM) and live-culture mageu (LCM) batches determined by quantitative PCR. (b) Relative bacterial abundance of SBM and LCM determined by 16S rRNA gene sequencing.

Nutritional analysis of SBM and LCM confirmed a similar composition of both products, although LCM had slightly higher energy, protein, carbohydrate, sugar, fibre and sodium contents (online Supplementary Table 1). When measuring absolute 16S rRNA gene copy number by broad-range quantitative PCR from a subset of mageu batches (SBM *n* 16, LCM *n* 23), we found that SBM batches had lower total 16S rRNA gene copy numbers per μl gDNA, with 11/16 (68·75 %) of the SBM batches tested having < 10 copies/μl gDNA, compared with only 3/23 (13·0 %, *P* < 0·001) of LCM batches tested, likely reflecting pasteurisation of the SBM. In agreement, the median 16S rRNA gene copy number per μl gDNA extracted from SBM (0, IQR 0–53) was significantly lower as compared with LCM (median 5679, IQR 313–36 988, *P* < 0·001, Figure 2(a)).

The proportion of live *v*. dead bacteria in both products was not assessed in detail, although external laboratory assessment of the LCM indicated that the lactic acid bacteria count was > 300 000 CFU/g. However, we conducted selective bacterial culture on MacConkey agar (to enrich for *E. coli*) and BHI agar (to enrich for *Clostridium* spp.) to comply with South African National Standards. None of the SBM and LCM batches contained live *E. coli*, and only two SBM batches (2/66, 3·03 %) contained live non-*E. coli* species, while most LCM batches contain live non-*E. coli* species (58/73, 79·45 %, *P* < 0·0001), with a median of 1·8 × 10^5^ CFU/ml (IQR 1·3 × 10^5^ − 1·1 × 10^6^). Similarly, only 3/66 (4·55 %) batches of SBM contained live obligate anaerobic spore formers, while 27/66 (40·91 %) batches of SBM contained facultative spore-forming species (*P* < 0·0001). Although these results were obtained sfrom selective bacterial culturing, they, together with the quantitative PCR data, suggest that the total bacterial load in LCM was higher than that in pasteurised SBM.

16S rRNA gene sequencing of SBM (*n* 16) and LCM (*n* 23) samples revealed that microbial composition of SBM and LCM differed. The core bacterial taxa of SBM were *Lactobacillus* species, whereas *Clostridium sensu stricto 1*, *Koskonia cowanii* and *Bacillus anthracis* were core taxa in LCM (Figure 2(b)). In agreement, SBM had significantly higher CLR-transformed abundance of *Lactobacillus* species (*L. amylolyticus* and *L. delbruckeii*), *Ruminococcus bromi* and *Prevotella copri* than LCM, while LCM had higher CLR-transformed relative abundance of a wide range of bacterial taxa when compared with SBM, including *Clostridium sensu stricto beijernickii*, *Bacillus anthracis* and *Klebsiella* species (*K. quasipneumoniae*, *K. pneumoniae* and *K. variicola*) (online Supplementary Table 2).

### Consumption of both store-bought mageu or live-culture mageu was generally safe and did not affect maternal BMI

Few AEs occurred in this study, and the frequency was comparable between groups. Throughout the intervention, three women in the no mageu group experienced fever, headache and an upper respiratory tract infection (each *n* 1); four women assigned to SBM experienced abdominal pain/bloating, diarrhoea, headaches and worsening of pre-existing seizures (each *n* 1); and three women assigned to LCM experienced abdominal pain/bloating, diarrhoea and fever (each *n* 1). Abdominal pain/bloating and diarrhoea were possibly related to mageu consumption, while the remainder were considered unrelated. There were no significant differences in BMI cross-sectionally at week 10 (end of intervention) by randomisation group, nor any significant changes in BMI from pre- (week 4) to post-intervention (week 10) (online Supplementary Table 3).

### Mageu consumption resulted in higher plant protein intake

Results from the 24-h recalls showed that during the intervention period the total energy intake in all three groups was slightly below the estimated energy requirement^(48)^ of adult women, protein intake was above the RDA for adult women^(49)^, while fibre intake was below the RDA. Further, intake of Ca^(50)^, Mg, vitamin C, vitamin E and folate^(49)^ was below the estimated adequate requirement for all three groups (Table 2).

Cross-sectional comparisons showed that plant protein intake differed between groups (adj. *P* = 0·041) during the intervention period (Table 2), with a significantly higher plant protein intake in the SBM group as compared with the no mageu group. No significant differences between groups were observed in the intake of total energy, fibre, total protein, Ca, Mg, Zn, Fe, vitamins A, C and E, B vitamins, beta-carotene and flavonoids. As energy intake strongly correlated with several nutrient indices including protein, fibre, Ca and Fe (online Supplementary Fig. 1), we adjusted for energy intake in subsequent analyses.

Assessment of frequency of intake of the ten food FAO^(27)^ groups included in the daily monitoring sheets showed that diets across all groups were dominated by starches and flesh foods (meat, poultry and fish) during the intervention period, while intake of dairy products, pulses, nuts and seeds was rare (online Supplementary Table 4). Reported intake of grains, roots and tubers (adj. *P* = 0·05) and meat, poultry and fish (adj. *P* = 0·03) was significantly higher in the no mageu group compared with the LCM group (but not the SBM group) (online Supplementary Table 4). The median dietary diversity score derived from the 10 food groups was 5·0 (IQR 4·0–5·3) for the SBM group, 4·0 (IQR 4·0–4·5) for the no mageu group and 5·0 (IQR 4·5–5·5) in the LCM group, with no significant difference between the groups (*P* = 0·28).

### Live-culture mageu increased Shannon gut microbiota diversity, while store-bought mageu and no mageu did not

**Figure 3.**
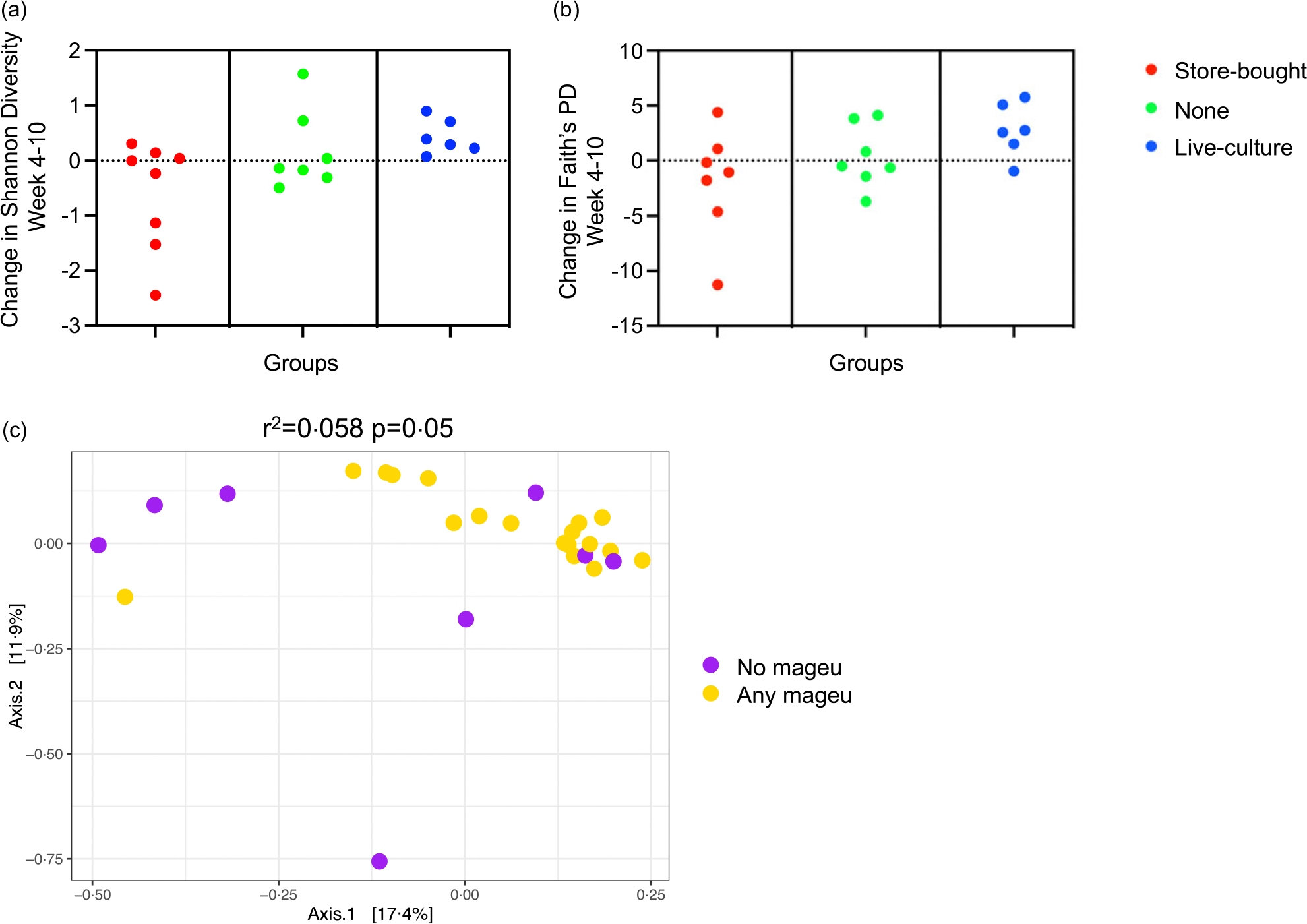
Stool microbiota diversity of women assigned to store-bought, none or live-culture mageu. (a), (b) Difference in *α*-diversity from week 4 to week 10 using Shannon index (a) and Faith’s phylogenetic diversity (PD) (b). (c) Beta-diversity using Bray–Curtis distances at week 15 compared by mageu use. Any mageu use includes women randomised to store-bought and live-culture mageu.

There were 139 stool samples from forty-four women with available data for analyses, after excluding eleven samples that were filtered out for low read count. The primary outcome was the change in stool *α*-diversity using Shannon index or Faith’s PD after 6 weeks of intervention (week 4 to week 10). Twenty-one participants had paired samples available for this analysis. Shannon index increased significantly in the LCM group, while this was not the case for the SBM and no mageu groups (Figure 3(a), *P* = 0·02). Similarly, Faith’s PD significantly increased in the LCM group, which was not seen in the SBM and no mageu groups (Figure 3(b), *P* = 0·04). Employing linear mixed models for repeated measures, Shannon index positively increased from week 4 to 15 (estimate 0·12, SE 0·05, *P* = 0·02) in the LCM group (but not the SBM group) when compared with no mageu users, but Faith’s PD was not statistically different during this period (estimate 0·21, SE 0·18, *P* = 0·23) (online Supplementary Table 5).

Bray–Curtis distances did not differ significantly between groups at week 4 postpartum (pre-intervention, *P* = 0·76), nor at week 10 (*P* = 0·27) or week 15 (*P* = 0·18) (online Supplementary Fig. 2). However, when combining LCM and SBM groups and comparing to the no mageu group, any mageu use accounted for a small but significant portion of the variability of community composition at week 15 (*r*^2^ = 0·058, *P* = 0·050, Figure 3(c)).

**Figure 4.**
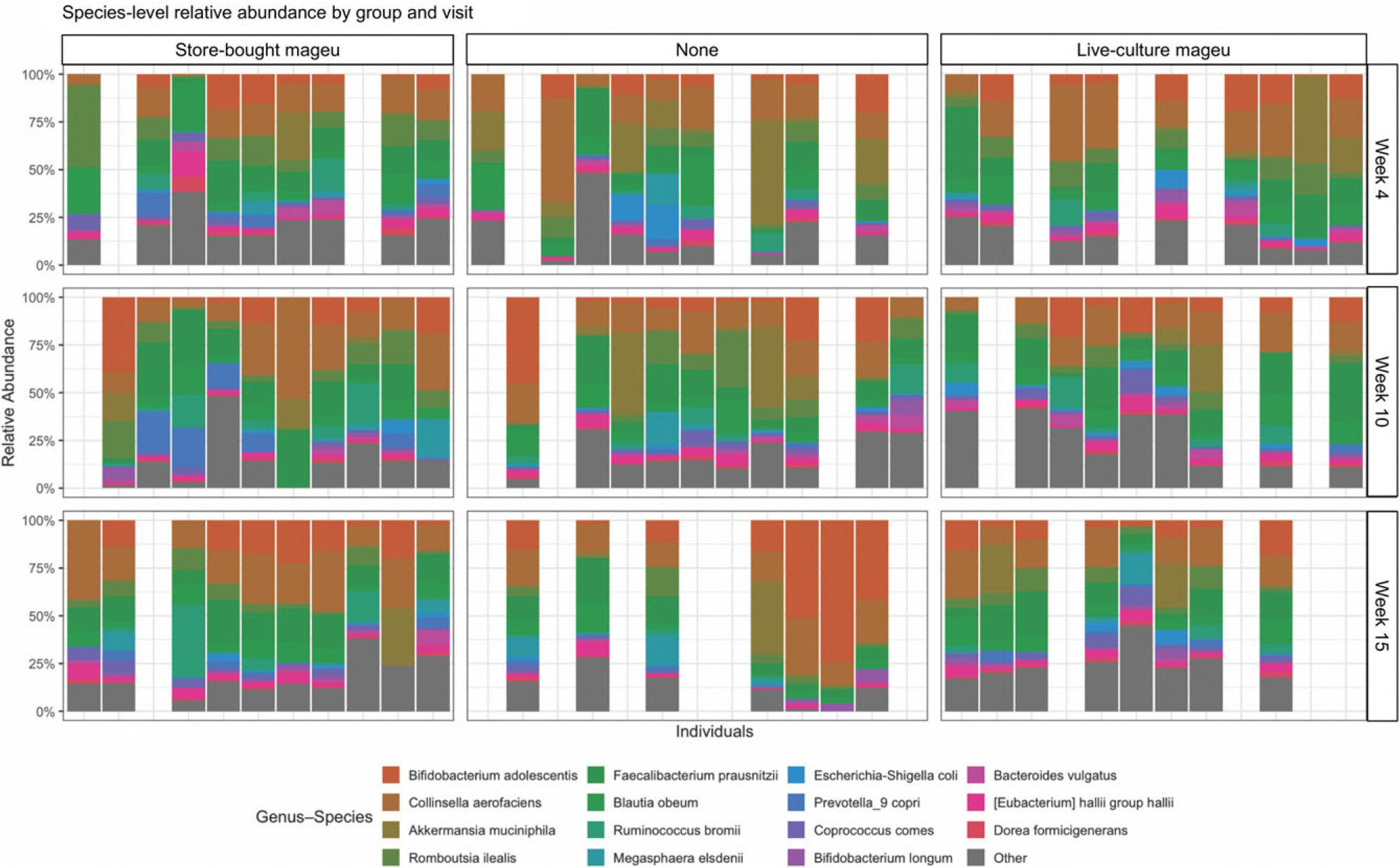
Stool microbiota species-level relative abundances of women assigned to store-bought, none or live-culture mageu. Rows represent the time points at which samples were collected (weeks 4, 10 and 15), and each column corresponds to a participant.

Relative abundance assessments of the maternal gut microbiota at weeks 4, 10 and 15 showed that a diverse range of species was present in samples from all three groups, including *Bifidobacterium adolescentis*, *Collinsella aerofaciens*, *Akkermansia muciniphilia*, *Romboutsia ilealis*, *Faeclibacterium prausnitzii*, *Blautia obeum*, *Ruminococcus bromii*, *Megasphaera elsdenii*, *Escherichia coli*, *Prevotella 9 copri*, *Coprococcus comes, Bifidobacterium longum*, *Bacteriodes vulgatus*, *Eubacterium halii* and *Dorea formicigenerans* (Figure 4). In presence–absence analyses between groups, forty unique taxa were identified in the LCM group at week 10 and 20 unique taxa at week 15. Overall prevalence of these taxa was low (11–33 %), with none consistently shared across participants. Two species showed moderate prevalence: *Lactococcus lactis* at week 10 (44 % in the LCM group) and *Ruminococcus torques* at week 15 (50 % in the LCM group). However, neither taxon appeared among the top 15 taxa in the relative abundance profiles (Figure 4), nor did they impact the community composition (online Supplementary Fig. 2).

**Figure 5.**
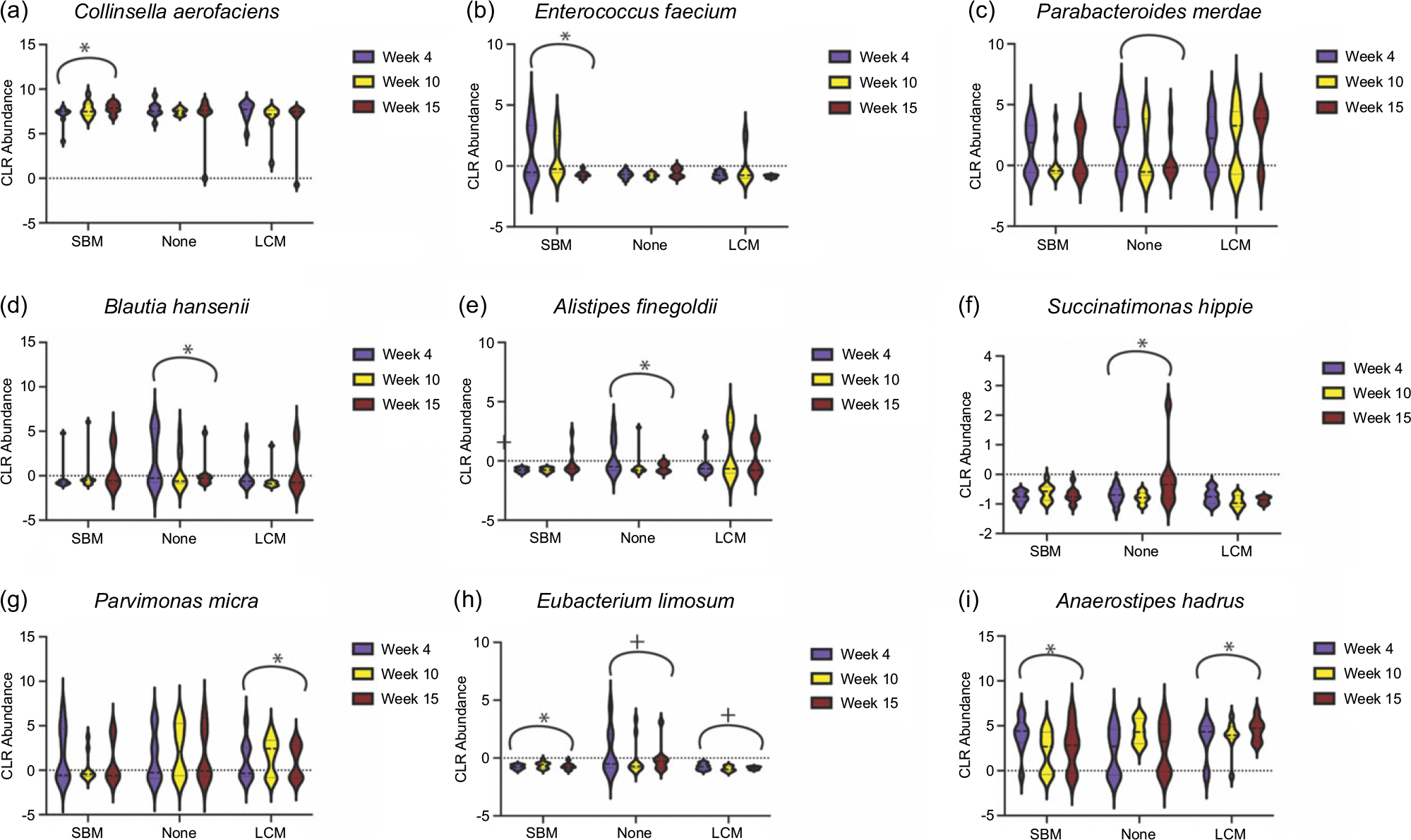
Change of bacterial taxa over time in gut microbiota of women assigned to store-bought (SBM), none or live-culture mageu (LCM). Violin plots showing bacteria, including (a) *Collinsella aerofaciens*, (b) *Enterococcus faecium*, (c) *Parabacteroides merdae*, (d) *Blautia hansenii*, (e) *Alistipes finegoldii*, (f) *Succinatimonas hippie*, (g) *Parvimonas micra*, (h) *Eubacterium limosum* and (i) *Anaerostipes hadrus*, identified by linear mixed models to have groups that had a change in centre log ratio (CLR) abundance over time after adjusting for total energy intake. *Adjusted *P*-value < 0·05, + adjusted *P*-value < 0·01.

After adjusting for total energy intake and accounting for repeated measures, there were significant changes in CLR abundance of taxa over time dependent on intervention group (Figure 5). *Collinsella aerofaciens*, one of the most abundant bacteria in the gut microbiota of this cohort, significantly increased in the SBM group with no changes in LCM and no mageu groups (Figure 5(a), estimate 0·10, *P* = 0·02). *Enterococcus faecium* significantly declined over time in the SBM group with no changes in LCM and no mageu groups (Figure 5(b), estimate –0·12, *P* = 0·003). *Parabacteroides merdae* significantly declined in the no mageu group (Figure 5(c), estimate –0·11, *P* = 0·03). *Blautia hansenii* increased non-significantly the SBM group (Figure 5(d), estimate 0·11, *P* = 0·064) and declined significantly over time in the no mageu group (estimate –0·18, *P* = 0·04). *Alistipes finegoldii* decreased in the no mageu group (Figure 5(e), estimate –0·099, *P* = 0·04). *Succinatimonas hippei* increased in the no mageu group with no significant changes in the SBM or LCM groups (Figure 5(f), estimate 0·035, *P* = 0·02). *Parvimonas micra* increased with time in the LCM group with no significant changes in the no mageu or SBM groups (Figure 5(g), estimate 0·026, *P* = 0·04). *Eubacterium limosum* increased in the SBM group (Figure 5(h), estimate 0·043, *P* = 0·01) and declined in the no mageu and the LCM groups (no mageu estimate –0·067, *P* = 0·0009 and LCM estimate –0·012, *P* = 0·001). Anaerostipes hadrus declined in the SBM group (Figure 5(i), estimate –0·13, *P* = 0·04) and increased in the LCM group (estimate 0·10, *P* = 0·04). Overall, these results indicate that LCM consumption increases gut *α*-diversity and that mageu intake may have induced taxonomic shifts in the gut microbiota of postpartum mothers.

### Live-culture mageu did not affect host inflammatory markers but decreased circulating ferritin compared with no mageu users

We measured faecal calprotectin in maternal stool and lipocalin-2 and myeloperoxidase in maternal plasma at weeks 4 and 10 as markers of intestinal inflammation. There were no significant differences cross-sectionally after completion of the intervention at week 10, nor in change between pre- (week 4) to post-intervention (week 10) (online Supplementary Table 3).

We measured various markers of systemic inflammation at weeks 4 and 10 (online Supplementary Table 3). In cross-sectional comparisons, there were no significant differences in TNF-*α*, IL-1*β*, IL-6, CRP or *α*−1-acid glycoprotein between groups at completion of the intervention (week 10), nor when comparing the change between weeks 4 and 10 (online Supplementary Table 3). However, ferritin levels differed significantly between groups at week 10 (adj. *P* = 0·023) and were significantly lower in the LCM group compared with the no mageu group (median 34·8 (IQR 32·8–45·1) μg/l *v*. 65·9 (IQR 55·9–77·4) μg/l, adj. *P* = 0·026) (online Supplementary Table 3).

### Mageu use did not affect iron and nutritional markers

Although ferritin can be an acute-phase reactant, it can also be a marker of iron sufficiency^(51)^. However, we found no differences in iron levels or sTfR between arms at week 10 (online Supplementary Table 3). Furthermore, total iron intake tended to be higher in the mageu groups than in the no mageu group (Table 2). There were also no differences in vitamin B_12_, vitamin D_3_, Tg or RBP4 between groups at week 10, nor when comparing the change in nutritional markers between weeks 4 and 10 (online Supplementary Table 3).

### System analysis revealed bacterial, inflammation and nutritional signatures unique to women randomised to mageu compared with no mageu

**Figure 6.**
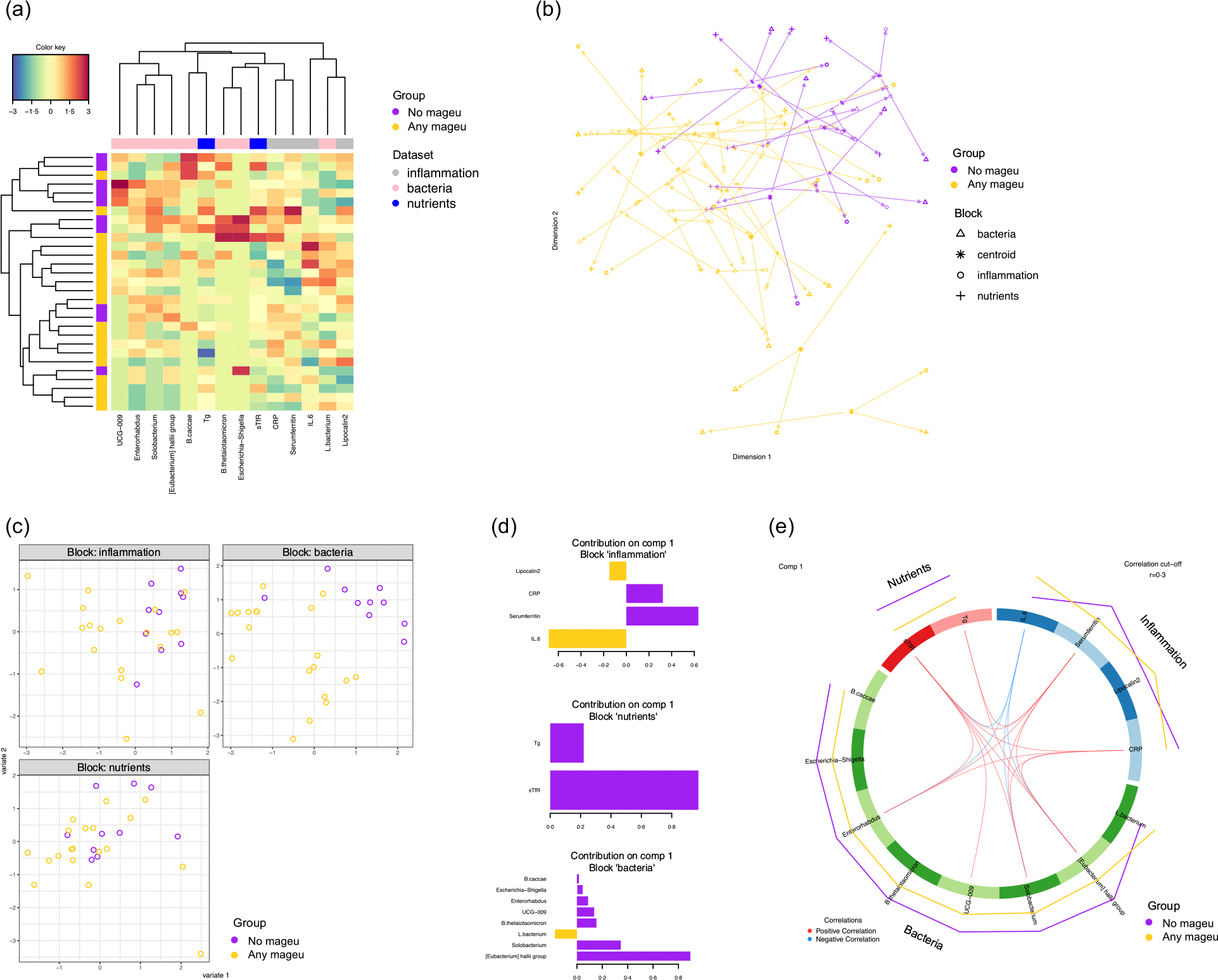
Integration of inflammation, nutrient and bacterial profiles at week 10 in women consuming any mageu or none. (a) Clustered Image Map for the variables selected by multiblock sPLS-DA performed on the inflammation, nutrient and bacterial datasets. Samples are presented in rows and selected features in columns. The colour key shows the range of correlation values. (b) Arrow plot from multiblock sPLS-DA. Samples of the data blocks inflammation, nutrients and bacteria are plotted into the space spanned by the first two components of the model. The length of the arrows indicates the distance of each sample from the centroids of both datasets. Short distances indicate high levels of agreement between dataset blocks, while longer distances indicate higher variability. (c) Sample plots from sPLS-DA performed on the inflammation, nutrient and bacterial datasets. Individual samples are projected onto the first two latent components from the model and coloured according to group to show the degree of agreement between the different blocks and the discriminative ability of each dataset. (d) Loading plot for the variables selected by multiblock sPLS-DA on component 1. The most important variables (according to the absolute value of their coefficients) are ordered from bottom to top for each of the data blocks. Colours indicate the group for which the median expression value is the highest for each feature. (e) Circos plot showing the correlations among the selected biomarkers. Red lines represent a positive correlation. Only correlations above a threshold cut-off of 0·3 were included. The outer purple (none) and orange (any) lines represent mageu use. sPLS-DA, sparse partial least squares discriminant analysis; DIABLO; Data Integration Analysis for Biomarker discovery using Latent cOmponents.

Finally, we integrated the inflammatory and nutritional markers and microbiota data to identify biomarkers that were distinct between any mageu (SBM and LCM) *v*. no mageu users at week 10. Sparse partial least squares discriminant analysis performed on the inflammation, nutrient and bacterial datasets from week 10 demonstrated that there was some clustering based on mageu use (none *v*. any) (Figure 6(a)). In the arrow plot (Figure 6(b)), each arrow represents a participant. Short, similarly oriented arrows indicate agreement among inflammation, nutrient and bacterial profiles, while longer arrows reflect greater between-dataset variability. Samples from none *v*. any mageu users occupy different regions of the plot, showing partial group separation, but the long arrows suggest weak correspondence across datasets at the participant level. Accordingly, the sample plot (Figure 6(c)) shows little separation between mageu groups in the nutrient and inflammation blocks but clearer separation in the bacterial block. In agreement, the identified loadings (Figure 6(d)) that differentiated the two groups best included four inflammatory markers (IL-6, ferritin, CRP and lipocalin-2), two nutritional markers (sTfR and Tg) and eight bacterial taxa (*Eubacterium hallii* group, *Solobacterium*, *Lachnospiraceae* bacterium, *Bacteroides thetaiotaomicron*, *Ruminococcaceae* UCG-009, *Enterorhabdus*, *Escherichia*-*Shigella* and *Bacteroides caccae*). Most selected bacterial, nutritional and inflammatory markers were lower among mageu users compared with non-users, except for IL6, lipocalin-2 and relative abundance of *Lachnospiraceae* (Figure 6(e)). The most important markers that distinguished mageu *v*. no mageu users were ferritin, sTfR and *E. hallii*, all of which were lower among women consuming mageu compared with non-users, and IL-6, which was higher (Figure 6(d)). Ferritin correlated positively with Tg, sTfR, *Enterorhabdus*, *Solobacterium* and *E. halli*, while IL-6 correlated negatively with sTfR, *Enterorhabdus*, *Solobacterium* and *E. halli* (Figure 6(e)).

## Discussion

Diet is a major driver of gut microbiome composition and function^(5,6,52,53)^ and subsequent systemic inflammation,^(8,9)^ both of which have been described as underlying causes of many metabolic diseases such as obesity, hypertension and diabetes^(6,54)^. Maternal nutrition is crucial not only for the mothers’ health but also for that of their children. Many people in southern Africa face challenges of poverty and inadequate access to a diverse array of foods^(55)^. Assessing the impact of a locally acceptable fermented food on maternal gut and immune health provides significant insight into the feasibility of local foods to improve the health of a population at risk. Our results utilising a store-bought pasteurised mageu v. a traditional live-culture preparation of mageu *v*. no mageu assessed the impact on the postpartum lactating mother’s gut microbiota, nutritional status and inflammation.

Overall, our intervention was found to be feasible. Our inoculum produced a product with a wide array of microbes. While batch-to-batch effects were present, the core genera were *Clostridium sensu stricto 1*, *Koskonia* and *Bacillus* in line with previously described mageu composition by sequencing^(20,56)^. In contrast, the SBM had its standard inoculum of lactobacilli consistently identified by sequencing. Bacterial culture and quantitative PCR suggest that the bacterial load of LCM was generally higher than that of SBM. SBM might however still contain inactivated microbial cells or cell components, with or without metabolites, which may confer health benefits – potentially serving as a ‘postbiotic’^(57)^. This demonstrates that LCM can reliably be produced without pathogen contamination and contains more complex microbial communities than pasteurised SBM.

Postpartum women enrolled in this study showed good compliance with the intervention, suggesting that women found consumption of mageu to be indeed acceptable and feasible. Women in the no mageu group reported increased consumption of starchy foods compared with mageu users, indicating that mageu users were likely substituting normal intake of other starchy foods with mageu. This supports the cultural acceptability of mageu. Furthermore, dietary intake in the three groups was comparable, with the only difference being the significantly higher intake of plant protein among women consuming mageu, regardless of whether this was store-bought or live-culture product, as compared with non-consumers. Importantly, there were no significant changes in BMI from the beginning to the end of the intervention period, which may be linked to the finding that the total energy intake in all three groups was in line with the estimated energy requirement and did not differ significantly between the groups. This is not surprising as participants received dietary counselling to ensure that energy intake during the study period was aligned with optimal caloric intake. The dietary diversity score for the intervention period was found to be poor and on the lower cut-off of 5 out of 10 for good diversity^(27)^ but did not differ significantly between groups. The trend towards a poorer dietary diversity may explain the low intake of fibre, Ca, Mg, vitamin C, vitamin E and folate in all three groups. Inadequate intake of Ca, vitamin C and folate in lower socio-economic communities in the Western Cape of South Africa is not uncommon^(58)^.

We found that stool microbial diversity was higher and increased during the study in women randomised to LCM, significantly more so than in mothers receiving SBM or no mageu. These results encouragingly suggest that LCM might be an acceptable, cheap, easily implementable and already available avenue to improve gut health in lactating mothers. Furthermore, the change in Shannon index was maintained until week 15 in the LCM group, although this was not true for Faith’s PD. It is thus unclear how long these changes may last or if they require continued mageu consumption to be sustained. Improved gut microbial diversity has been associated with decreased risk of obesity^(59,60)^ and insulin resistance^(61)^, suggesting that mageu may improve metabolic health in women. However, as we recorded no change in BMI over the 6-week intervention period, a larger sample and longer intervention period may be necessary to investigate these potential health benefits.

We found changes in faecal bacterial taxa indicative of risk for metabolic diseases that differed between mageu randomisation groups and over time. For example, using centred log ratio transformed abundance of taxa, *Blautia hanseii* was increased in the SBM group and decreased in the no mageu group. *B. hansenii* is negatively associated with visceral fat accumulation^(62)^ and, in animal models, was found to mitigate the effects of a high-fat diet^(63)^. LCM increased *Anaerostipes hadrus* relative abundance, a bacterium known to degrade fructooligosaccharides and to increase SCFA^(64)^. In contrast, *A. hadrus* was decreased with time in the SBM group. One study found *A. hadrus*, which harbours a composite inositol catabolism-butyrate biosynthesis pathway, to be associated with low host metabolic risk assessed by BMI and abdominal circumference^(65)^. The no mageu group had a decrease in *Parabacteroides merdae*, which has been associated with branched-chain amino acid catabolism and decreased atherosclerosis^(66)^. Overall, these findings suggest that LCM may alter the evolving postpartum gut microbiota favouring taxa associated with metabolic health.

The change in gut microbial diversity was not accompanied by a general decrease in inflammation, neither for intestinal nor for systemic markers measured. This is inconsistent with a previous meta-analysis of randomised controlled trials that found fermented foods to lead to a reduction of systemic TNF-*α* levels^(16)^. However, the same authors found that intake of fermented foods did not improve circulating CRP and IL-6^(16)^, which is in line with our findings. The relatively short duration of the intervention and small sample size might not have been sufficient to see modulation of the immune system through changes in the stool microbiota. Further, in this group of women without comorbidities who were recently pregnant (a state of relatively low inflammation), it may be more difficult to see changes in inflammation. We found that ferritin was significantly lower in the LCM group than the no mageu group at week 10. Ferritin is an acute-phase reactant that coordinates cellular defense against oxidative stress and inflammation along with transferrin and its receptor^(51)^. Ferritin levels are also markers of iron sufficiency^(51)^. As we did not see differences between groups in transferrin receptor and iron levels, low ferritin indeed might have been a marker of low inflammation, with its decrease coinciding with an increase in stool diversity. As differences in stool microbial diversity and ferritin levels were only observed in the LCM group, this could point towards the superiority of live-culture over pasteurised mageu in its ability to improve gut and immune health of lactating women.

Integration of nutritional, bacterial and inflammatory datasets revealed that the most variance between mageu and no mageu use was driven by ferritin, sTfR and *E. halli*, all higher with no mageu use, and IL-6, which was lower among no mageu users. IL-6 plays an important role in the activation of host defense after infection and injury. However, excessive or sustained production of IL-6 is involved in various diseases^(67)^. A recent systematic review found no effects of fermented food consumption on IL-6^(16)^, and larger cohorts are necessary to further elucidate the relationship between IL-6 and mageu consumption. As mentioned above, ferritin can act as a marker of both acute inflammation and iron sufficiency. When tissue iron availability is low, membrane receptors are upregulated to take more iron into the cells, resulting in increased serum sTfR levels^(68)^, making high sTfR a marker of iron deficiency. Mageu may therefore dampen acute-phase inflammation (i.e. lower CRP and ferritin) and stabilise iron stores (i.e. lower sTfR). *E. hallii* is considered a key species within the intestinal trophic chain with the potential to impact metabolic balance as well as the gut microbiota/host homeostasis by the formation of SCFA^(69)^. Butyrate and other SCFA, through different mechanisms, inhibit intestinal inflammation, maintain the intestinal barrier and modulate gut motility^(70)^.

This study was designed to explore the effects of a locally acceptable fermented food on both the gut microbiota and inflammation in postpartum women – a population group whose nutritional status is central to the development of their children, given that they are exclusively breastfeeding. Although this study provided critical preliminary and encouraging insights into the effect of mageu consumption on gut microbiota composition and immune status, we acknowledge several limitations. This study included a small sample size of mostly overweight women, which limits power and our ability to generalise results. We used BMI as a surrogate measure of obesity and acknowledge that BMI might be an inaccurate individual measure of health^(71)^. The intervention phase was limited to 6 weeks, and a longer intervention period might have resulted in more profound changes in gut microbiota and inflammation. Participants in this urban area were generally healthy, albeit overweight, with a risk of low intake of some micronutrients, and future studies could explore the effect of mageu on gut and immune health among people diagnosed with inflammatory conditions. Strengths included the study of lactating women, a group that is usually excluded from research. Furthermore, the study included a no mageu control arm and comparisons between LCM and SBM, increasing the practicality of the findings. Lastly, the study design also included a washout phase, allowing us to generate a similar ‘baseline’ diet profile among all participants.

In conclusion, we showed that LCM might have beneficial effects on the gut and immune health of women. Given the local context and relevance of these findings for maternal and infant nutrition, this should be explored in a larger cohort. Assessment of maternal breastmilk and infant gut microbiota, immune status and overall health would provide significant insight into the usability of a plant-based, local fermented food to improve maternal and infant health outcomes.

## Supplementary Material

1

For supplementary material/s referred to in this article, please visit https://doi.org/10.1017/S0007114526106862.

## Figures and Tables

**Table 1.** Cohort characteristics of the women included in the analysis

Characteristic	All (*n* 44)		SBM (*n* 13)		None (*n* 16)		LCM (*n* 15)	
	*n*	%	*n*	%	*n*	%	*n*	%
Age at enrolment, years								
Median	27		24		27		31	
IQR	23–33		21–28		23–32		27–33	
Formal housing, *n* (%)	21	47·7	6	46·2	9	56·3	6	40·0
Unemployed, *n* (%)	41	93·2	11	84·6	16	100	14	93·3
BMI at week 4*								
Median	29·6		29·8		30·5		28·7	
IQR	26·6–34·9		27·8–34·3		25·9–35·3		27·4–34·5	
Primiparous, *n* (%)	15	34·1	5	38·5	6	37·5	3	20·0
Running water inside, *n* (%)	37	84·1	12	92·3	14	87·5	12	80·0
Married, *n* (%)	11	25·0	3	23·1	3	18·8	5	33·3

SBM, store-bought mageu; LCM, live-culture mageu; IQR, interquartile range.

Characteristics are described as median (IQR) for continuous variables and *n* (%) for categorical variables.

**Table 2.** Energy and nutrient dietary intake during the intervention period based on 24-h recall data

	EER/EAR/AI/RDA	SBM (*n* 12)		None (*n* 11)		LCM (*n* 11)		Adj. *P*
		Mean	SD	Mean	SD	Mean	SD	
Energy (kcal)	Median 9939, IQR 9511–10 607 (EER)^(48)^	9308·90	1131·41	8228·02	1410·19	9006·62	1224·42	0·12
Carbohydrate (g)	-	319·13	54·37	276·14	69·89	314·61	57·31	0·10
Total protein (g)	Median 63, IQR 55–71 (RDA)^(49)^	74·08	8·52	65·52	11·16	71·41	13·20	0·10
Plant protein (g)	-	30·77	5·30	24·94	5·03	29·01	5·70	**0**·**04**
Animal protein (g)	-	36·97	4·92	35·65	7·77	37·03	7·95	0·87
Total fat (g)	-	62·90	4·73	59·75	6·28	61·20	5·84	0·41
Saturated fat (g)	-	17·05	2·10	16·01	2·41	16·82	2·80	0·57
Monounsaturated fat (g)	-	21·27	2·28	20·26	3·38	20·10	2·75	0·56
Polyunsaturated fat (g)	-	17·53	0·43	17·28	0·43	17·43	0·44	0·41
Trans fat (g)	-	0·46	0·34	0·52	0·47	0·49	0·40	0·95
Cholesterol (g)	-	221·54	135·03	200·18	130·34	270·96	211·55	0·58
Total fibre (g)	25 g (AI)^(49)^	17·82	3·34	15·02	3·59	17·12	3·44	0·15
Added sugar (g)	-	20·83	24·18	30·55	27·37	23·23	25·22	0·65
Ca (mg)	800 (EAR)^(50)^	391·62	117·84	309·71	144·44	429·64	140·26	0·12
Mg (mg)	350 (EAR)^(50)^	251·32	49·72	206·97	51·36	233·44	57·27	0·15
Fe (mg)	6 (EAR)^(49)^	14·75	3·22	11·90	2·74	14·29	4·47	0·14
Zn (mg)	9·4 (EAR)^(49)^	12·25	2·08	10·72	1·80	11·18	2·12	0·19
Vitamin A (mcg)	500 (EAR)^(49)^	675·59	158·07	571·91	180·53	677·94	249·60	0·37
Vitamin C (mg)*	60 (EAR)^(49)^	19·90	0·00	19·90	0·00	19·90	0·00	-
Vitamin E (mg)	12 (EAR)^(49)^	9·50	0·14	9·41	0·15	9·48	0·12	0·25
Vitamin B_1_ (Thiamine) (mg)	0·9 (EAR)^(49)^	1·68	0·42	1·37	0·31	1·55	0·38	0·16
Vitamin B_2_ (Riboflavin) (mg)	0·9 (EAR)^(49)^	1·17	0·47	0·97	0·29	1·22	0·55	0·40
Niacin (mg)*	11 (EAR)^(49)^	27·90	0·00	27·90	0·00	27·90	0·00	-
Vitamin B_6_ (mg)	1·1 (EAR)^(49)^	3·58	0·41	3·35	0·35	3·48	0·42	0·38
Folate (mcg)	320 (EAR)^(49)^	2·68	1·60	2·39	1·66	2·47	1·25	0·90
Vitamin B_12_ (mcg)	2 (EAR)^(49)^	2·68	1·60	2·39	1·66	2·47	1·25	0·90
Beta-carotene (mg)	-	671·34	790·70	364·68	343·89	513·76	557·31	0·48
Flavonoids (mg)	-	103·70	90·27	108·32	196·53	54·44	58·34	0·55

EER, estimated energy requirement; EAR, estimated average requirement; AI, adequate intake; SBM , store-bought mageu; LCM , live-culture mageu; IQR, interquartile range.

The adjusted mean (SD) is shown. Adj. *P* = adjusted *P*-values. Statistics were calculated using an ANOVA test with Dunn’s tests for multiple comparisons. EER = median (IQR) estimated energy requirement calculated using the following formula: (575·77 – (7·01 × age in years) + (6·60 × height in cm) + (12·14 × weight in kg at baseline/week 4)) × 4·2 kJ (assumption of low physical activity). RDA = median (IQR) RDA calculated using the following formula: 0·8 × weight in kg at baseline (week 4). EAR = estimated average requirements for females 19 and older.

* The F-statistic for these models was < 1, resulting in the between-participant variance being zero.

## Abbreviations:

BHI: brain heart infusion
CFU: colony-forming units
CLR: centred log ratio
CRP: C-reactive protein
DIABLO: Data Integration Analysis for Biomarker discovery using Latent cOmponents
IQR: interquartile range
LCM: live-culture mageu
PD: phylogenetic diversity
SBM: store-bought mageu
