# Supplementary material for: Culturally acceptable fermented grain may improve gut health in South African postpartum mothers in a randomised trial
Source: Br J Nutr. Author manuscript; Available in PMC 2026 Jun 8. (PMC13243706; doi:10.1017/S0007114526106862)
Supplement: 1 [file NIHMS2155859-supplement-1.docx]

| **Supplementary Table 1.** Nutritional analysis of mageu products per serving (500 ml). | | |
| --- | --- | --- |
|  | **SBM** | **LCM** |
| **Energy [kj]** | 569 | 855 |
| **Protein [g]** | 2.0 | 5.2 |
| **Carbohydrates [g]** | 30.0 | 43.8 |
| **Sugar [g]** | 14.5 | 21.9 |
| **Fat [g]** | 1 | <0.05 |
| **Saturated fat [g]** | 0 | 0 |
| **Trans fat [g]** | 0 | 0 |
| **Polyunsaturated fat [g]** | 0 | 0 |
| **Monaunsaturated fat [g]** | 0 | 0 |
| **Cholesterol [mg]** | 0 | <5 |
| **Fibre [g]** | 1.0 | 2.6 |
| **Sodium [mg]** | 10.0 | 28.2 |
| **Potassium [mg]** | 0 | 0 |
| Values were obtained by an external laboratory for LCM and the manufacturer of SBM. SBM = store-bought mageu, LCM= live-culture mageu | | |

| **Supplementary Table 2.** Univariable linear regression models of various taxa center log ratio transformed abundances and mageu type (store-bought versus live-culture). All results were corrected for multiple comparisons. | | |
| --- | --- | --- |
| **Taxa** | **Estimate (Standard Error)** | **Adjusted p-value** |
| ***Lactobacillus amyloticus*** | -9.8 (1.3) | <0.001 |
| ***Lactobacillus delbruckeii*** | -7.1 (1.2) | <0.001 |
| ***Clostridium sensu stricto beijerinckii*** | 2.6 (0.9) | 0.03 |
| ***Bacillus anthracis*** | 3.9 (0.7) | <0.001 |
| ***Klebsiella quasipneumoniae*** | 2.8 (0.8) | 0.002 |
| ***Klebsiella pneumoniae*** | 5.7 (0.7) | <0.001 |
| ***Klebsiella variicola*** | 3.0 (0.9) | 0.03 |
| ***Ruminococcus bromii*** | -2.5 (0.8) | 0.04 |
| ***Prevotella 9 copri*** | -2.2 (0.7) | 0.007 |
| Positive estimates indicate that the taxa was increased amongst the live-culture mageu compared to store-bought mageu. | | |

| **Supplementary table 3.** BMI, inflammation, iron and nutritional data from the three intervention groups. | | | | | | | | | |
| --- | --- | --- | --- | --- | --- | --- | --- | --- | --- |
|  |  | **Week 10 cross-sectional comparison** | | | | **Difference Week 4-Week 10 comparison** | | | |
|  |  | **SBM** | **None** | **LCM** | **Adj. p** | **SBM** | **None** | **LCM** | **Adj. p** |
|  |  | **n=12** | **n=12** | **n=11** |  | **n=12** | **n=11^#^** | **n=11** |  |
|  | **BMI** | 30.00  [27.90, 34.90] | 31.26  [25.35, 37.20] | 27.90  [25.97, 32.33] | 0.46 | 0.19 [0.10, 0.58] | 0.00 [-0.69, 0.46] | 0.20 [0.08, 0.43] | 0.53 |
| **Intestinal inflammation** | **Faecal calprotectin (ng/mL)** | 55.88  [25.93, 231.93] | 113.18  [67.44, 243.34] | 178.44  [5.71, 440.94] | 0.90 | 4.38  [-52.07, 113.88] | 131.82  [34.98, 249.51] | 111.85  [102.11, 965.53] | 0.28 |
|  | **Lipocalin-2 (ng/mL)** | 120.62  [104.24, 129.16] | 104.83  [72.49, 149.72] | 134.38  [103.63, 162.15] | 0.37 | 12.09  [-14.59, 34.66] | -7.67  [-16.16, -1.53] | -2.82  [-24.96, 48.94] | 0.79 |
|  | **MPO (ng/mL)** | 934.97  [704.84, 1093.04] | 973.98  [719.53, 1374.90] | 1068.14  [814.15, 1407.05] | 0.73 | 82.87  [-187.98, 225.62] | -152.08  [-434.05, 362.42] | -21.62  [-386.77, 610.81] | 0.83 |
| **Systemic inflammation** | **TNF-α (pg/mL)** | 15.14  [12.54, 16.26] | 16.39  [13.22, 20.51] | 17.34  [13.38, 19.34] | 0.36 | 0.48 [-0.52, 1.32] | -0.15 [-1.28, 2.58] | 0.70 [-2.51, 6.01] | 0.99 |
|  | **IL-1β(pg/mL)** | 5.65 [4.11, 6.84] | 6.60 [5.24, 7.75] | 5.82 [5.16, 7.40] | 0.50 | -0.17 [-0.42, 0.76] | -0.24 [-0.44, 0.40] | 0.20 [-0.17, 1.93] | 0.53 |
|  | **IL-6(pg/mL)** | 3.39 [1.84, 4.75] | 2.87 [2.31, 4.46] | 3.14 [2.82, 6.67] | 0.75 | -0.34 [-0.93, 0.48] | -0.25 [-1.52, 0.46] | 0.34 [-0.38, 3.22] | 0.27 |
|  | **CRP (mg/L)** | 4.31 [1.99, 6.30] | 4.60 [1.73, 6.40] | 2.37 [0.90, 6.29] | 0.88 | -0.49 [-2.00, 0.11] | -0.30 [-0.62, 2.32] | -0.49 [-3.14, 0.57] | 0.48 |
|  | **AGP (µg/L)** | 1.31 [1.12, 1.44] | 1.19 [1.14, 1.49] | 1.32 [1.21, 1.60] | 0.52 | 0.03 [-0.19, 0.06] | 0.04 [-0.08, 0.12] | 0.00 [-0.17, 0.09] | 0.75 |
| **Iron status** | **Ferritin (µg/L)** | 40.19  [21.27, 59.95] | 65.87  [55.91, 77.40] | 34.82  [32.84, 45.11] | **0.02** | -7.43  [-11.67, 2.68] | -8.23  [-17.83, 15.53] | -3.96  [-10.18, 2.35] | 0.88 |
|  | **Iron (µm/L)** | 12.05  [9.02, 15.65] | 13.90  [9.65, 16.47] | 13.60  [10.35, 15.50] | 0.78 | 1.35 [-2.20, 3.90] | -1.50 [-3.80, 0.35] | 0.00 [-2.40, 2.15] | 0.28 |
|  | **sTfR (mg/L)** | 4.46 [4.00, 5.28] | 4.87 [4.44, 5.67] | 4.61 [3.72, 5.22] | 0.63 | -0.52 [-0.78, 0.00] | -0.34 [-0.81, 0.51] | 0.14 [-0.13, 0.61] | 0.28 |
| **Nutritional status** | **Vitamin B12**  **(pmol/L)** | 375.00  [325.75, 440.25] | 366.50  [330.00, 546.00] | 436.00  [388.00, 474.50] | 0.61 | 26.50  [-9.00, 40.25] | 18.00  [11.00, 106.50] | 27.00  [-31.50, 73.00] | 0.72 |
|  | **Vitamin D3 (ng/ml)** | 58.70  [51.75, 74.80] | 64.70  [47.85, 74.10] | 63.80  [57.90, 76.05] | 0.79 | 6.50 [1.15, 10.80] | 5.00 [-0.75, 12.85] | 4.80 [-0.15, 10.05] | 0.94 |
|  | **Tg (µg/L)** | 10.43  [6.12, 16.74] | 11.66  [7.18, 15.11] | 11.19  [9.38, 13.97] | 0.79 | 0.76 [0.37, 3.51] | -0.44 [-2.47, 2.98] | 3.04 [0.82, 3.84] | 0.21 |
|  | **RBP4 (µmol/L)** | 1.50 [1.15, 1.78] | 1.55 [1.40, 1.83] | 1.79 [1.24, 1.85] | 0.82 | -0.03 [-0.11, 0.11] | 0.00 [-0.18, 0.07] | -0.01 [-0.14, 0.16] | 0.87 |
| Median [IQR] is reported and Kruskal-Wallis test used for comparisons between groups, with Dunn’s test for multiple comparison adjustment. SBM = store-bought mageu, LCM = live-culture mageu. BMI = body mass index, MPO = myeloperoxidase, CRP = C-reactive protein, AGP = ⍺1-acid glycoprotein, sTfR = soluble transferrin receptor, Tg = thyroglobulin, RBP4 = retinol binding protein 4.  ^#^n=1 had no week 4 samples available. Adj. p = adjusted p-values. | | | | | | | | | |

| **Supplementary Table 4.** Frequency of intake of the 10 food groups during the intervention period based on daily monitoring sheets. | | | | |
| --- | --- | --- | --- | --- |
| **Frequency / week, median [IQR]** | **SBM (n=12)** | **None (n=11)** | **LCM (n=11)** | **Adj. p** |
| **Grains, roots and tubers** | 10.17 [7.67, 14.00] | 14.33 [13.25, 15.58] | 7.17 [2.67, 14.50] | **0.05** |
| **Pulses** | 0.75 [0.54, 1.58] | 0.33 [0.08, 0.42] | 0.83 [0.42, 1.33] | 0.17 |
| **Nuts and seeds** | 0.58 [0.29, 1.54] | 0.17 [0.08, 0.92] | 0.50 [0.33, 0.67] | 0.64 |
| **Dairy** | 0.08 [0.00, 0.50] | 0.83 [0.50, 1.67] | 0.50 [0.08, 0.75] | 0.07 |
| **Flesh foods (meat, poultry, fish)** | 6.58 [4.00, 8.88] | 8.33 [6.92, 8.75] | 3.00 [2.17, 6.08] | **0.02** |
| **Eggs** | 3.00 [1.79, 3.92] | 2.00 [1.42, 2.92] | 1.67 [1.17, 3.00] | 0.38 |
| **Dark green leafy vegetables** | 1.75 [1.46, 2.71] | 1.50 [0.58, 2.42] | 1.50 [1.00, 2.00] | 0.51 |
| **Other vitamin A-rich fruits and vegetables** | 2.50 [1.63, 3.29] | 1.33 [0.67, 3.50] | 2.33 [1.42, 3.33] | 0.61 |
| **Other vegetables** | 1.83 [1.17, 2.21] | 1.83 [1.67, 3.00] | 1.67 [1.08, 3.00] | 0.64 |
| **Other fruits** | 2.17 [1.00, 2.71] | 1.67 [1.25, 2.00] | 1.17 [0.58, 2.42] | 0.53 |
| The frequency of intake of the 10 food Food and Agriculture Organization^(27)^ groups was assessed in the daily monitoring sheets. Adj. p = adjusted p-values. P-values were adjusted for multiple comparisons using Dunn’s test. Statistics were calculated using a Kruskal-Wallis test with Dunn’s tests for multiple comparisons. | | | | |
|  | | | | |

| **Supplementary Table 5.** Linear mixed model depicting Shannon alpha-diversity and Faith’s phylogenic diversity from from weeks 4 to 15. | | | | |
| --- | --- | --- | --- | --- |
|  | **Shannon** | | **Faith’s** | |
| **Variable** | **Estimate (Standard Error)** | **p-value** | **Estimate (Standard Error)** | **p-value** |
| **Visit** | -0.067 (0.37) | 0.06 | -0.11 (0.13) | 0.41 |
| **Arm SBM** | -0.15 (0.52) | 0.77 | -0.56 (1.8) | 0.76 |
| **Arm LCM** | -0.62 (0.53) | 0.24 | -0.35 (1.9) | 0.85 |
| **Visit*Arm SBM** | 0.051 (0.049) | 0.31 | 0.059 (0.17) | 0.73 |
| **Visit*Arm LCM** | 0.12 (0.050) | **0.02** | 0.21 (0.18) | 0.23 |
| Participant ID was the random effect, and fixed effects were visit, intervention arm, and visit*arm. No mageu was the reference group. SBM = store-bought mageu, LCM = live-culture mageu. | | | | |


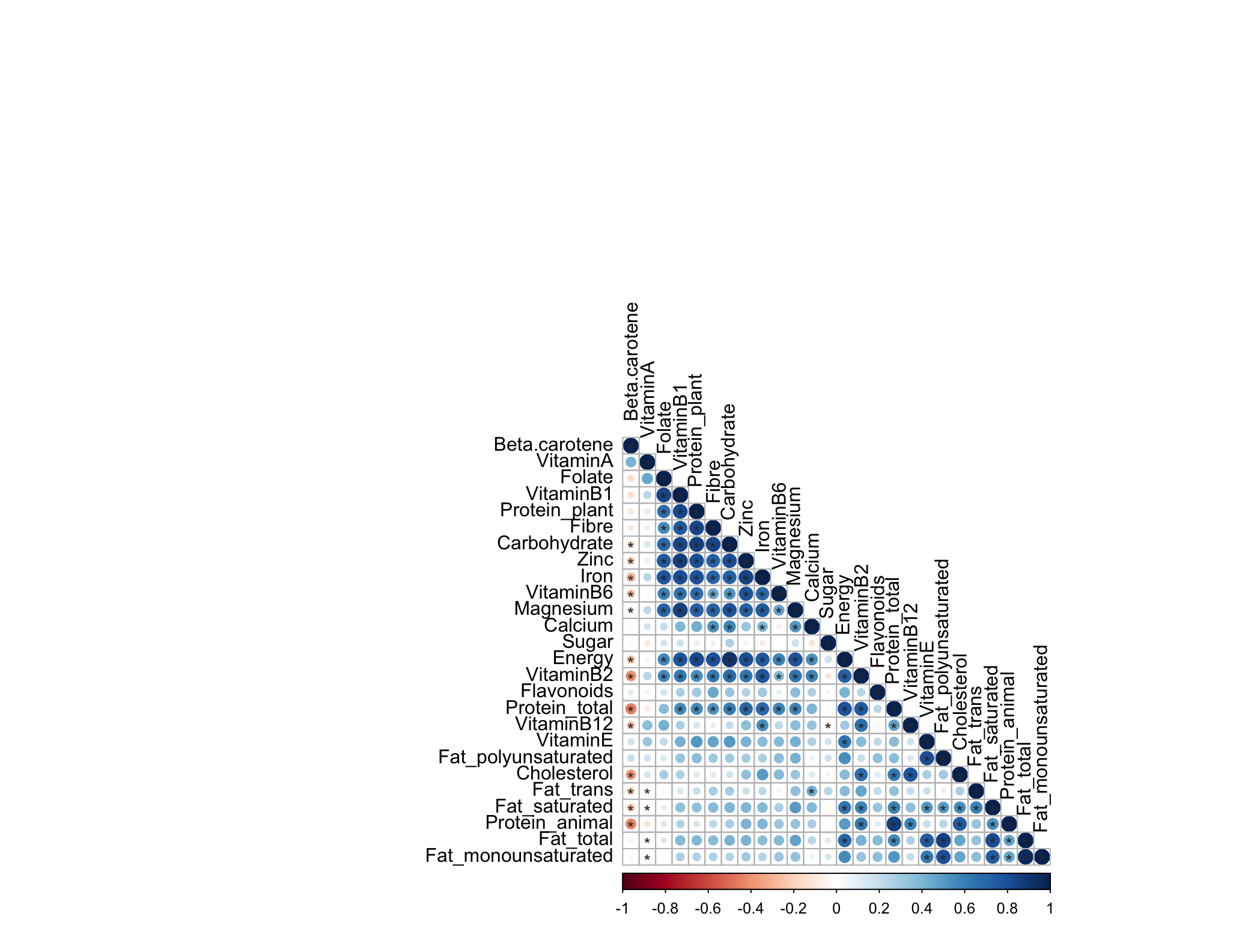


**Supplementary Figure 1.** Correlations between energy and nutrient intake. The color scale indicates directionality of Pearson correlations (red = negative, blue = positive correlation). * indicates adjusted p < 0.01.

**Supplementary Figure 2**. Gut microbiota beta-diversity using Bray-Curtis distances at weeks 4 (A), 10 (B), and 15 (C).
